# ThermoMixer-Aided Endpoint Quaking-Induced Conversion (EP-QuIC) Permits Faster Sporadic Creutzfeldt-Jakob Disease (sCJD) Identification than Real-Time Quaking-Induced Conversion (RT-QuIC)

**DOI:** 10.1128/JCM.00423-18

**Published:** 2018-06-25

**Authors:** Robert Vendramelli, Angela Sloan, Sharon L. R. Simon, Debra Godal, Keding Cheng

**Affiliations:** a National Microbiology Laboratory, Public Health Agency of Canada, Winnipeg, MB, Canada; b Department of Human Anatomy and Cell Sciences, College of Medicine, University of Manitoba, Winnipeg, MB, Canada; Rhode Island Hospital

**Keywords:** EP-QuIC, RT-QuIC, prions, sCJD

## LETTER

We recently reported a method called endpoint quaking-induced conversion (EP-QuIC) (1), which is modified from real-time quaking-induced conversion (RT-QuIC) (2–4), to aid in antemortem diagnosis of sporadic Creutzfeldt-Jakob disease (sCJD). In both QuIC reactions, the presence of minute amounts of misfolded prion protein induces the conversion of exogenous recombinant prion protein (rPrP) during intermittent shaking at 42°C (900 rpm for 90 s and rest for 30 s for each round), which is monitored fluorometrically (1–4). RT-QuIC uses a plate reader for the shaking step and needs approximately 90 h of incubation. However, shaking performed at high speed for extended periods of time increases the wear on the hardware, resulting in the need for repeated servicing of the system (1). On the other hand, EP-QuIC utilizes a benchtop shaking incubator (a ThermoMixer), and relative fluorescent units (RFU) are quantified at the beginning (RFU_initial_; time zero) and end (RFU_final_, time 90 h) of the reaction. While our report demonstrated that the diagnostic sensitivities and specificities of the two methods were in almost perfect agreement, it was observed that EP-QuIC reactions elicited higher RFU values at the 90-h reaction time and that the thresholds determined for the two versions of the test were distinctly different (1). This prompted us to investigate the dynamics of these two QuIC platforms.

**FIG 1:**
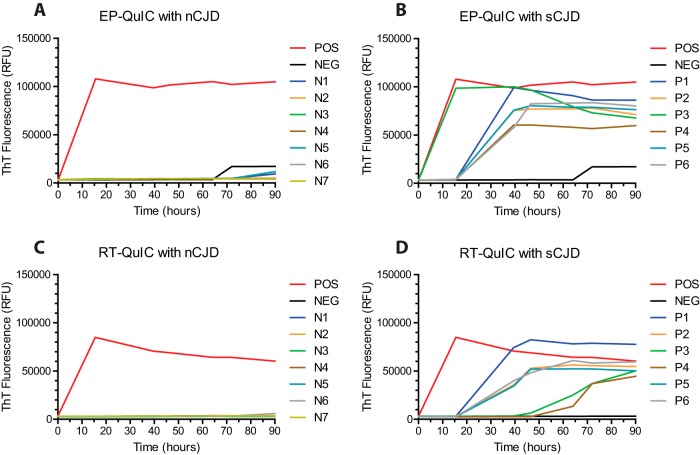
EP-QuIC and RT-QuIC dynamics of well-confirmed sCJD-negative (N) samples (*n* = 7) and sCJD-positive (P) samples (*n* = 6). The negative control (NEG) was artificial CSF, and the positive control (POS) was a 10^−2^ dilution of product as seed (PAS). Both EP-QuIC and RT-QuIC were performed on the basis of a method described in reference 1 (please see the main text for further details).

Control material and diagnostically defined human cerebrospinal fluid (CSF) samples were tested. The negative control was artificial CSF (Harvard Apparatus), and the positive control was a 10^−2^ dilution of product as seed (PAS), made from the positive QuIC reaction products from an autopsy-confirmed sCJD patient (5). Tests performed on these control samples were confirmed to be as highly reproducible as earlier tests (1). Table S1 in the supplemental material and graphs in Fig. 1 show results of testing randomly selected representative clinical samples; 6 autopsy-confirmed sCJD CSF samples and 7 diagnostically confirmed non-CJD (nCJD) CSF samples were analyzed by EP-QuIC and RT-QuIC as previously described (1), with additional manual reads of EP-QuIC plates performed at 16-, 40-, 47-, 64-, 72-, and 90-h intervals. The EP-QuIC reactions were found to take place faster, with all sCJD samples exhibiting a saturated signal at 47 h, while RT-QuIC signals from some sCJD samples were still increasing at 90 h. Single-well autoaggregation was seen with one negative control after 64 h and with two nCJD samples after 72 h on EP-QuIC and with one nCJD sample at 90 h on RT-QuIC. When the cutoff threshold at 47 h was set at a value of 2 (RFU_final_/RFU_initial_) for EP-QuIC, instead of the value of 4 required for a 90-h reaction time (1), its sensitivity and specificity were same as those seen with RT-QuIC at 90 h when the same threshold was used (both were 100% sensitive and specific) (see Table S2A). Validation tests performed on 51 CSF samples (27 sCJD, 24 nCJD) confirmed this observation (see Table S2B).

Many factors affect QuIC reactions (6), and there has been no international standard determined for this test. The International Conference on Harmonisation (ICH) protocol for analytical method validation recommended by Health Canada and the U.S. FDA accepts a signal twice the level of noise (background) for qualitative analyses (7, 8). We highly recommend using EP-QuIC with daily reading for less than 48 h; if an average reading is twice the background level, a positive reaction should be called.

## Supplementary Material

Supplemental material

## References

[1] Cheng K, Vendramelli R, Sloan A, Waitt B, Podhorodecki L, Godal D, Knox JD 2016 Endpoint quaking-induced conversion: a sensitive, specific, and high-throughput method for antemortem diagnosis of Creutzfeldt-Jacob disease. J Clin Microbiol 54:1751–1754. doi:10.1128/JCM.00542-16. 27076662 PMC4922112

[2] McGuire LI, Peden AH, Orru CD, Wilham JM, Appleford NE, Mallinson G, Andrews M, Head MW, Caughey B, Will RG, Knight RS, Green AJ 2012 Real time quaking-induced conversion analysis of cerebrospinal fluid in sporadic Creutzfeldt-Jakob disease. Ann Neurol 72:278–285. doi:10.1002/ana.23589. 22926858 PMC3458796

[3] Wilham JM, Orru CD, Bessen RA, Atarashi R, Sano K, Race B, Meade-White KD, Taubner LM, Timmes A, Caughey B 2010 Rapid end-point quantitation of prion seeding activity with sensitivity comparable to bioassays. PLoS Pathog 6:e1001217. doi:10.1371/journal.ppat.1001217. 21152012 PMC2996325

[4] McGuire LI, Poleggi A, Poggiolini I, Suardi S, Grznarova K, Shi S, de Vil B, Sarros S, Satoh K, Cheng K, Cramm M, Fairfoul G, Schmitz M, Zerr I, Cras P, Equestre M, Tagliavini F, Atarashi R, Knox D, Collins S, Haik S, Parchi P, Pocchiari M, Green A 2016 Cerebrospinal fluid real-time quaking-induced conversion is a robust and reliable test for sporadic Creutzfeldt-Jakob disease: an international study. Ann Neurol 80:160–165. doi:10.1002/ana.24679. 27130376 PMC4982084

[5] Cheng K, Sloan A, Waitt B, Vendramelli R, Godal D, Simon SLR, O'Neil J, Carpenter M, Jackson D, Eastlake J, Mallinson G, Knox JD 2018 Altered rPrP substrate structures and their influence on real-time quaking induced conversion reactions. Protein Expr Purif 143:20–27. doi:10.1016/j.pep.2017.10.007. 29031681

[6] Orrú CD, Hughson AG, Groveman BR, Campbell KJ, Anson KJ, Manca M, Kraus A, Caughey B 2016 Factors that improve RT-QuIC detection of prion seeding activity. Viruses 8:E140. doi:10.3390/v8050140. 27223300 PMC4885095

[7] Health Canada. 2015 Q2(R1) Validation of analytical procedures: text and methodology. http://www.hc-sc.gc.ca/dhp-mps/prodpharma/applic-demande/guide-ld/ich/qual/q2r1-eng.php Accessed 18 November 2016.

[8] FDA. 1996 Guidance for industry: Q2B validation of analytical procedures. http://www.fda.gov/downloads/drugs/guidancecomplianceregulatoryinformation/guidances/ucm073384.pdf Accessed 18 November 2016.

