# Supplementary material for: ThermoMixer-Aided Endpoint Quaking-Induced Conversion (EP-QuIC) Permits Faster Sporadic Creutzfeldt-Jakob Disease (sCJD) Identification than Real-Time Quaking-Induced Conversion (RT-QuIC)
Source: J Clin Microbiol. 2018 Jun 25;56(7):e00423-18. doi: 10.1128/JCM.00423-18 (PMC6018336; doi:10.1128/JCM.00423-18)
Supplement: Supplemental material [file supp_56_7_e00423-18__index.html]

ThermoMixer-Aided Endpoint Quaking-Induced Conversion (EP-QuIC) Permits Faster Sporadic Creutzfeldt-Jakob Disease (sCJD) Identification than Real-Time Quaking-Induced Conversion (RT-QuIC) — Supplemental material 

# ThermoMixer-Aided Endpoint Quaking-Induced Conversion (EP-QuIC) Permits Faster Sporadic Creutzfeldt-Jakob Disease (sCJD) Identification than Real-Time Quaking-Induced Conversion (RT-QuIC)

## Supplemental material

- Supplemental file 1 -

  Tables S1A (EP-QuIC average readings on artificial CSF and seven non-CJD patient samples), S1B (EP-QuIC average readings on PAS and six positive sCJD patient samples), S1C (RT-QuIC average readings on artificial CSF and seven non-CJD patient samples), S1D (RT-QuIC average readings on PAS and six positive sCJD patient samples), S2A (Method verification and data analysis on 13 confirmed patient samples), and S2B (Method validation and data analysis on 51 patient samples)

  PDF, 259K
