# Supplementary material for: ThermoMixer-Aided Endpoint Quaking-Induced Conversion (EP-QuIC) Permits Faster Sporadic Creutzfeldt-Jakob Disease (sCJD) Identification than Real-Time Quaking-Induced Conversion (RT-QuIC)
Source: J Clin Microbiol. 2018 Jun 25;56(7):e00423-18. doi: 10.1128/JCM.00423-18 (PMC6018336; doi:10.1128/JCM.00423-18)
Supplement: Supplemental material [file JCM.00423-18_zjm999095990s1.pdf]

Supplementary Table 1 (S1)

S1-A, EP-QuIC average readings on artificial CSF and seven non-CJD patient samples

Hours	-ve A-CSF	N1	N2	N3	N4	N5	N6	N7
0	3127	3139	3143	3404	3195	3156	3098	3173
16	3453	3979	3951	4456	4145	3958	4011	3928
40	3599	4505	4499	4335	4485	4419	4259	4296
47	3639	4578	4623	4390	4493	4438	4273	4346
64	3610	4675	4834	4367	4478	4644	4253	4336
72	17035	4831	4895	4360	4497	4723	4186	4283
90	17153	9388	5002	4427	4443	11874	4028	4353

S1-B, EP-QuIC average readings on PAS and six positive sCJD patient samples

Hours	+ve PAS 10-2	P1	P2	P3	P4	P5	P6
0	3158	3245	3344	3062	3108	3132	3323
16	107886	4139	4189	98598	3715	4017	4196
40	98676	99210	75257	100108	60481	75695	57847
47	101688	96473	77117	96782	60416	80424	82454
64	105099	90784	77162	79479	58010	78780	83146
72	102207	86341	77993	73088	56712	78783	83538
90	104956	86131	71299	67649	59854	76304	80215

S1-C, RT-QuIC average readings on artificial CSF and seven non-CJD patient samples

Hours	-ve A-CSF	N1	N2	N3	N4	N5	N6	N7
0	3109	3173	3180	2647	2671	2680	3032	3103
16	2967	3225	3196	2717	2767	2669	3032	3080
40	3029	3577	3482	2867	2997	2965	3331	3258
47	3012	3604	3539	2949	3215	2975	3336	3288
64	3090	3650	3597	3136	3237	3074	3468	3403
72	3139	3684	3667	2983	3195	3057	3492	3433
90	3170	3669	3710	2956	3158	3096	5922	3454

S1-D, RT-QuIC average readings on PAS and six positive sCJD patient samples

Hours	+ve PAS 10^{-2}	P1	P2	P3	P4	P5	P6
0	3192	3185	3138	3180	2592	2594	2647
16	84927	3220	3221	3219	2531	2668	2718
40	70632	74459	35795	3465	2650	34512	39961
47	68903	82497	52890	6468	2629	51945	48186
64	64340	78219	56253	25052	13437	52345	60901
72	64191	78802	55585	37090	36972	52226	58252
90	60367	77744	54651	50127	44572	50183	59607

Supplementary Table 2 (S2)

S2-A Method verification and data analysis on 13 confirmed patient samples*

Method	EP-QuIC			RT-QuIC	
Cutoff	2	2	4	2	2
Time @read	47	90	90	47	90
TN	7	5	7	7	6
FP	0	2	0	0	0
TP	6	6	6	5	6
FN	0	0	0	1	0
Total	13	13	13	13	13
Sensitivity (%)	100	100	100	83	100
Specificity (%)	100	71	100	100	100

*7 non-CJD and 6 sCJD. TN, true negative; FP, false positive; TP, true positive; FN, false negative

S2-B Method validation and data analysis on 51 patient samples*

Method	EP-QuIC			RT-QuIC	
Cutoff	2	2	4	2	2
Time @read	47	90	90	47	90
TN	23	23	23	24	24
FP	1	1	1	0	0
TP	27	27	27	1	24
FN	0	0	0	17	3
Total	51	51	51	51	51
Sensitivity (%)	100	100	100	37	89
Specificity (%)	96	96	96	100	100

*24 non-CJD and 27 sCJD. TN, true negative; FP, false positive; TP, true positive; FN, false negative
